# Capsaicin Improves Systemic Inflammation, Atherosclerosis, and Macrophage-Derived Foam Cells by Stimulating PPAR Gamma and TRPV1 Receptors

**DOI:** 10.3390/nu16183167

**Published:** 2024-09-19

**Authors:** Danielle Lima Ávila, Weslley Fernandes-Braga, Janayne Luihan Silva, Elandia Aparecida Santos, Gianne Campos, Paola Caroline Lacerda Leocádio, Luciano Santos Aggum Capettini, Edenil Costa Aguilar, Jacqueline Isaura Alvarez-Leite

**Affiliations:** 1 Departamento de Bioquímica e Imunologia, ICB, Universidade Federal de Minas Gerais, Belo Horizonte 31270-901, Brazil; danilimavila@gmail.com (D.L.Á.); weslley.fernandes.braga@gmail.com (W.F.-B.); janayneluihan@gmail.com (J.L.S.); elandianutri@gmail.com (E.A.S.); paolaleocadio@yahoo.com.br (P.C.L.L.); edenilaguilar@gmail.com (E.C.A.); 2 Departamento de Farmacologia, ICB, Universidade Federal de Minas Gerais, Belo Horizonte 31270-901, Brazil; giannepcampos@gmail.com (G.C.); lucianocapettini@gmail.com (L.S.A.C.)

**Keywords:** atherosclerosis, capsaicin, foam cells, inflammation, TRPV1, PPARγ

## Abstract

Background: Capsaicin, a bioactive compound found in peppers, is recognized for its anti-inflammatory, antioxidant, and anti-lipidemic properties. This study aimed to evaluate the effects of capsaicin on atherosclerosis progression. Methods: Apolipoprotein E knockout mice and their C57BL/6 controls were utilized to assess blood lipid profile, inflammatory status, and atherosclerotic lesions. We also examined the influence of capsaicin on cholesterol influx and efflux, and the role of TRPV1 and PPARγ signaling pathways in bone marrow-derived macrophages. Results: Capsaicin treatment reduced weight gain, visceral adiposity, blood triglycerides, and total and non-HDL cholesterol. These improvements were associated with a reduction in atherosclerotic lesions in the aorta and carotid. Capsaicin also improved hepatic oxidative and inflammatory status. Systemic inflammation was also reduced, as indicated by reduced leukocyte rolling and adhesion on the mesenteric plexus. Capsaicin decreased foam cell formation by reducing cholesterol influx through scavenger receptor A and increasing cholesterol efflux via ATP-binding cassette transporter A1, an effect primarily linked to TRPV1 activation. Conclusions: These findings underscore the potential of capsaicin as a promising agent for atherosclerosis prevention, highlighting its comprehensive role in modulating lipid metabolism, foam cell formation, and inflammatory responses.

## 1. Introduction

Capsaicin (trans-8-methyl-*N*-vanillyl-6-nonenamide, C18H27NO3) is a prominent member of the capsaicinoids, a group of lipophilic and volatile compounds. Capsaicin (Cap) is the most pungent and abundant among the capsaicinoids [1]. The vanilloid ring in Cap has a high affinity for the transient receptor potential vanilloid subtype 1 (TRPV1), a non-selective cation channel widely distributed in various tissues. This receptor is highly expressed in neural and adipose tissue, hepatocytes, leukocytes, and endothelial cells [2]. As a polymodal receptor, TRPV1 responds to a broad spectrum of physical and chemical stimuli, with Cap being its primary exogenous ligand.

For decades, Cap has been extensively studied as an adjuvant for treating inflammatory diseases, particularly neuropathic pain. More recently, the potential effects of Cap on chronic non-communicable diseases have gained interest [3].

Capsaicin’s biological activities include analgesic, anesthetic, anti-inflammatory, antioxidant, and thermogenic effects, which are potentially beneficial in chronic diseases such as obesity, systemic arterial hypertension, diabetes mellitus, and dyslipidemias [4,5,6,7].

Regarding atherosclerosis, capsaicin intake can improve lipid profiles, reduce inflammation, and mitigate endothelial dysfunction, all of which are crucial factors in preventing or slowing the progression of atherosclerosis [8]. Therefore, our objective was to evaluate the role of Cap on risk factors associated with atherosclerosis using ApoE KO mice, a model highly susceptible to atherosclerosis. Specifically, we focused on its effects on plaque formation as well as its role in regulating intracellular cholesterol in macrophage-derived foam cells.

## 2. Materials and Methods

### 2.1. In Vivo Study

Forty 6–8 week-old female mice deficient in the apoprotein E (ApoE KO) and their wild type (WT) C57BL/6 controls (*n* = 18), originally from Jackson Laboratory and kept in the animal facility of the Federal University of Minas Gerais (Belo Horizonte, Brazil), were divided according to initial body weight into groups. The WT and ApoE-KO groups received a standard diet (AIN-96M) supplemented with 0.75% cholesterol, and WT + Cap and ApoE + Cap groups received the same diet supplemented with 0.015% Cap (Sigma, Ronkonkoma, NY, USA), as previously described [9,10]. Animals had ad libitum access to water and food, and were maintained under controlled conditions (temperature: 26–28 °C, light-dark cycles: 12 h) in collective cages (2–3 mice/cage) within the same animal facility room. Mice were housed in cages that were clearly labeled with the corresponding descriptions of their respective experimental groups to mitigate any potential dietary contamination. In the third week of the diet, animals underwent carotid occlusion surgery to accelerate atherosclerotic development. In the fifth experimental week, after overnight fasting, animals were anesthetized for blood collection and euthanized by exsanguination. Following perfusion with phosphate-buffered saline (PBS), liver, aorta, and carotid samples were collected. Weekly assessments included body weight and food intake for energy consumption and weight gain calculations. Liver and visceral adipose tissue weight were measured to calculate relative weight (tissue/body weight × 100). Biological samples were assigned unique codes to ensure that the researchers remained blinded to the specific group assignments of the samples, thereby preserving the integrity of the experimental design and minimizing bias in the analysis. This study was approved by the Ethics Committee of Animal Experimentation—UFMG (# 232/2016). Mice that presented any health issues (such as malocclusion due to incisors overgrowth) were excluded.

#### 2.1.1. Carotid Obstruction Surgery

Carotid obstruction was performed to accelerate atherosclerosis formation. The surgery procedure followed the method described by Nam et al. [11]. Briefly, animals were anesthetized (ketamine 80 mg/kg, xylazine 15 mg/kg) and maintained on a thermal blanket at 37 °C. The left carotid artery was exposed and delicately tied with surgical wire (Sil Silkam 3/0, B. Braun, Germany). Afterward, the region was sutured, and the mice were observed until complete recovery.

#### 2.1.2. Biochemical Determinations

Blood glucose, total cholesterol (TC), HDL cholesterol (HDL-c), and triglycerides (TG) were quantified using colorimetric assay kits (Labtest, Lagoa Santa, Brazil) and following the manufacturer’s recommendations. The atherogenic fraction, non-HDL cholesterol (non-HDLc), was calculated from the difference between TC and HDL-c.

#### 2.1.3. Quantification of the Atherosclerosis Area

The aortas were removed, cleaned, and stained with SUDAN IV, as previously described [12]. Image acquisition and analysis were performed using the Image-Pro Plus version 6.3 analyzer (Image-Pro Plus Software, Rockville, MD, USA). The results were presented as the percentage of the aorta area affected by fatty streak. Carotids were included in the Tissue Freezing Medium (Jung Tissue Freezing Medium; Leica Microsystems, Wetzlar, Germany) and were immediately frozen. After processing, slides were stained with Hematoxylin-Eosin (HE) for analysis using Image-Pro Plus software.

#### 2.1.4. Immunofluorescence

The carotid slides were fixed in acetone, permeabilized, and blocked with 0.1% Triton X-100 and 4% BSA in PBS for 1 h before overnight incubation with the primary antibodies (mouse CD36 anti-mouse, Santa Cruz, Dallas, TX, USA; rat MOMA anti-mouse, Bio-Rad Hercules, CA, USA diluted 1:200). After washing, slides were incubated with fluorescent secondary antibodies (rabbit FITC anti-mouse—Santa Cruz; goat Alexa Fluoride 647 anti-mouse—BD Pharmigen, San Diego, CA, USA) for 2 h, washed and covered with DAPI-containing mounting medium. The images were captured with a Nikon Eclipse Ti fluorescence microscope (Melville, NY, USA), and the intraplaque fluorescence intensity (FI) was analyzed using the ImageJ software 1.39. The content of CD36 and MOMA in the lesion was expressed as the fluorescence intensity (FI).

#### 2.1.5. Determination of NAG and MPO Activities

*N*-acetyl-beta-D-glucosaminidase (NAG) and myeloperoxidase (MPO) activities were measured to assess macrophage and neutrophil infiltrations, respectively, as previously described [13].

#### 2.1.6. Cytokine Determination

To evaluate inflammatory status, TNF and IFN-γ, IL-6, and IL-10 concentrations were detected in liver homogenate by ELISA using a commercial kit (Biolegend, San Diego, CA, USA) and following the manufacturer’s instructions.

#### 2.1.7. Intravital Microscopy of Mesenteric Plexus

Intravital microscopy was performed as described [14] to evaluate inflammation in an in vivo real-time manner. Mice were kept in a thermal blanket (37 °C) throughout the procedure. After anesthesia, animals were injected (IV) with 0.1 mL of Rhodamine 6 G (0.5 mg/mL) (Sigma Aldrich, St. Louis, MI, USA) for fluorescence visualization of leukocytes in mesenteric microcirculation. Afterward, the abdominal region was opened, and the intestinal loops were exposed for the mesenteric microcirculation visualization using a microscope Apotome 2 (Zeiss, Oberkochen, Germany) with a 100 × increase. Video captures were made for 10 min for each animal. The number of rolling or adhered cells was counted by delimiting an area in the vessel lumen of 200 μm × 250 μm for 1 min for each count. Cells remaining in the same place in the delimited area for at least 30 s were considered adhered. All analyses were performed in triplicate using the ZEN Blue software 2.1 (Zeiss). The results were expressed as the number of rolling cells or the number of adhered cells/min. After data acquisition, animals were euthanized under anesthesia, as described above.

### 2.2. In Vitro Studies

#### 2.2.1. Preparation of Dil-Oxldl

Native human LDL was isolated by gradient ultracentrifugation from the plasma of healthy donors and was isolated as described by Chung and colleagues [15]. LDL protein concentration was then determined by Lowry et al. [16]. LDL was labeled with DiI as previously described [17]. A solution of 1,1′-Dioctadecyl-3,3,3′,3′-tetramethylindocarbocyanine perchlorate (Dil, Sigma-Aldrich, USA) was prepared by mixing DiI 3 mg/mL of DMSO. This solution was added to the LDL (50 µL of Dil solution for each mg of LDL), followed by overnight incubation at 37 °C in the dark. After incubation, the labeled LDL was reisolated by ultracentrifugation, dialyzed against PBS, and filter sterilized as described above. The oxLDL-Dil preparation was carried out as described by Fernandes-Braga et al. [18]. Dil-LDL oxidation was obtained by incubation with 10 mM CuSO_4_ for 24 h at 4 °C in the dark. The oxidation was stopped by adding 0.5 M EDTA solution at a final concentration of 0.24 mM. Dil-oxLDL was subjected to new ultracentrifugation (50,000 rpm, 2:30 hs), new dialysis for 24 h, and filtration (0.22 µM) before use.

#### 2.2.2. Cell Cultures

Bone marrow-derived macrophage (BMDM): Cells were extracted in a sterile environment from bone marrow that was derived from the femur and tibia of C57BL/6 mice and deposited in an ice-cold Falcon tube containing RPMI medium without fetal bovine serum (FBS). After centrifugation at 200× *g* for 10 min at 40 °C, cells were resuspended in RPMI for differentiation into macrophages in a 5% CO_2_ atmosphere, at 37 °C, for 7 days, changing the medium on the 4th day. The cells were washed, and the adhered macrophages were released by ice-cold 10 mM EDTA solution. The released macrophages were placed in an RPMI medium supplemented with 5% FBS for experimental cultures.

#### 2.2.3. Solutions for Cell Cultures

The Capsaicin used was as follows: Cap stock solution at a concentration of 1 mM (*N*-vanillylnonanamide—Capsaicin—Sigma-Aldrich, purity ≥ 97%) diluted in 1.8% NaCl and ethanol, in a 1:1 ratio. This solution was then filtered through a 0.22 µm sterile filter and stored at 4 °C for up to 90 days.

Capsazepine (CZE) was diluted in DMSO to a final concentration of 1 mM (stock solution), filtered through a sterile 0.22 µm filter, and stored at −20 °C for up to 30 days. The concentration used in the tests was 10 µM, as previously described [19,20].

GW9662: The solution of the irreversible and selective PPARγ antagonist, GW9662 (Santa Cruz), was diluted to a concentration of 0.1 mM in DMSO. The solution was filtered (0.22 µm sterile filter) and stored at −20 °C for up to 60 days. The concentration used in the tests was 5 µM, as previously described [21,22].

Flow cytometry. The uptake of oxLDL-Dil, as well as the expression of the influx scavenger receptors CD36 and SRA, and those of efflux, ABCA1, and ABCG1, were detected on the macrophage surface by flow cytometry.

Cells were seeded in round-bottom 96-well plates with 5 × 10^5^ cells/well, in their respective medium supplemented with 5% SBF, to provide a minimum amount of HDL to enable cholesterol efflux.

Cells were pre-incubated without inhibitor or with 10 µM Capsazepine (TRPV1 antagonist) or 5 µM of GW9662 (PPARγ antagonist) for 1 h. After washing, the cells were then incubated with 1 µM or 5 µM of Cap for 2 h. After new washing to remove Cap, cells were incubated with oxLDL-Dil (final concentration of 50 μg/mL) for 4 h. Afterward, the cells were centrifuged and incubated for 30 min at room temperature with one of the following primary antibodies: mouse antimouse-CD36 (Santa Cruz, Dallas, TX, USA); goat antimouse SR-A (Santa Cruz); mouse antimouse-ABCA1 (Novus Biologicals, Centennial, CO, USA); or rabbit antimouse-ABCG1 (Novus Biologicals, Centennial, CO, USA). Cells were washed twice with PBS Wash before secondary antibodies were incubated for 30 min according to the primary antibody: goat anti-mouse Alexa Fluor 647 (Cell Signaling, Danvers, MA, USA), rabbit anti-goat Alexa 647, and donkey anti-rabbit CFL 647 (Santa Cruz). In the end, the cells were washed and fixed with 2% paraformaldehyde/PBS for 30 min at room temperature for subsequent readings on a FACS CaliburTM cytometer, using the FL-2 and FL-4 filters for Dil and the Alexa 647 probes, respectively. Data acquisition was carried out using the BD CellQuest Pro software 5.0.1, and the delimitation of the cell population was carried out using the size and internal complexity parameters (FSC and SSC parameters, respectively). Approximately 15,000–20,000 events were acquired per sample. Data were calculated as the percentage of fluorescence of populations distributed in two-dimensional dot plots. Data were analyzed using FlowJow v10.0.6 software (Tree Star Inc., Ashland, OR, USA).

### 2.3. Statistical Analysis

The sample size was calculated using the following online tool: https://clincalc.com/Stats/SampleSize.aspx (accessed on 10 September 2024). The calculation was based on the media of total cholesterol (*n* = 14/group), rolling cells (*n* = 6/group), and carotid lesion area (*n* = 9/group) and was based on the apo E KO mice study. The C57BL/6 mice sample size was based on total cholesterol (*n* = 7/group) and 6 mice were used for bone marrow-derived macrophage. The analyses were performed using the GraphPad Prism 8.0 software (GraphPad Software, San Diego, CA, USA). Data were verified for the null hypothesis of Gaussian distribution using the Kolmogorov–Smirnov test. The Rout test was performed to identify outliers (that were excluded from the analyses). The statistical comparison of three or more groups was performed by one-way ANOVA, followed by Tukey’s multiple comparisons post-test and Kruskal–Wallis test, with Dunns’ multiple comparisons post-test for parametric and non-parametric data, respectively. When Cap groups were compared to their respective controls, the unpaired Student’s *t*-test, and Mann–Whitney test were used for parametric and non-parametric data, respectively. The results were expressed as mean ± standard error (SE), with a significance level of 5%.

## 3. Results

### 3.1. Capsaicin Improves Weight, Reduces Visceral Fat, and Enhances Glycemia and Lipid Profile in ApoE KO Mice

**Figure 1:**
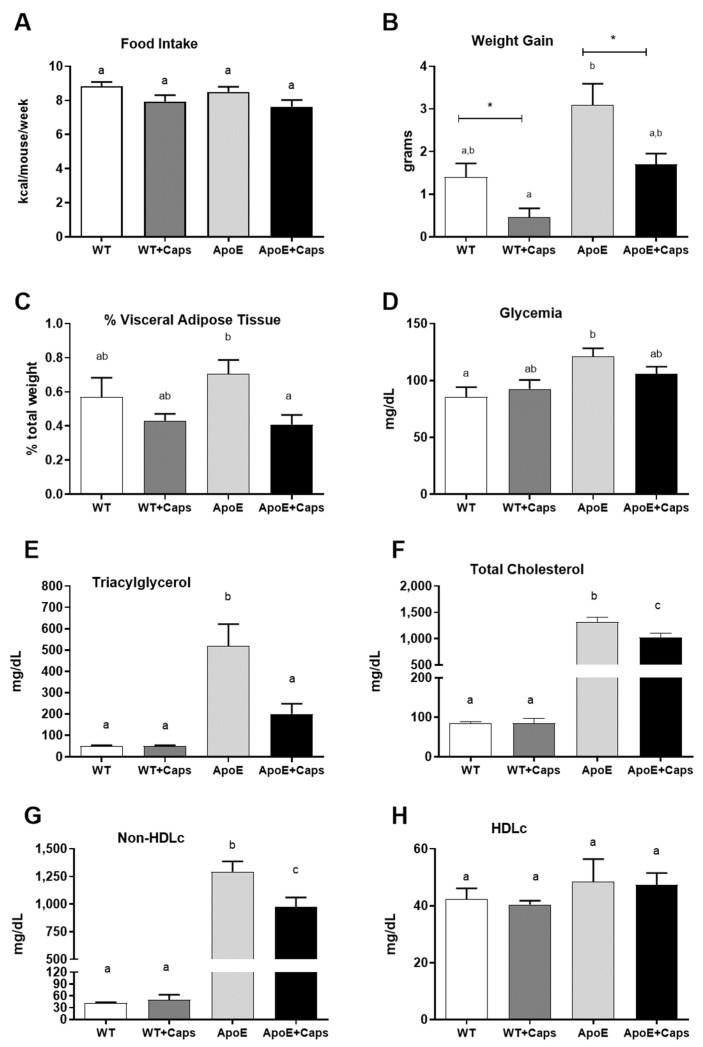
Effects of capsaicin on caloric intake, body composition, glycemia, and lipid profile in WT and ApoE-Deficient mice. Calorie intake (**A**), weight gain (**B**), fat percentage (**C**), glycemia (**D**), and lipid profile (**E**–**H**) of C57BL/6 and Apo E KO animals fed cholesterol-rich diet (AIN-93M + 0.75% cholesterol) without or with 0.015% capsaicin. Bars represent the mean, and vertical lines represent the standard error. Groups not sharing the same letter = statistically different. (*p* < 0.05). * T Student *t*-test compared with group without capsaicin. *N* mice = WT = 5, WT + CAP = 6, ApoE − KO = 11, ApoE + Cap = 10.

There was no difference in caloric intake between the evaluated groups (Figure 1A). Despite this, weight gain (Figure 1B) and visceral fat percentage (Figure 1C) were reduced by approximately 50% in ApoE KO animals receiving Cap compared to their controls. Glycemia was higher in the ApoE KO group than in the WT group (Figure 1D), while the Cap groups presented intermediated glycemia levels (Figure 1D).

Regarding lipid profile, concentrations of triglycerides (Figure 1E), total cholesterol (Figure 1F), and non-HDL cholesterol (*n*-HDLc, Figure 1G) were reduced in ApoE + Cap mice without a reduction in HDL cholesterol (Figure 1H). These results suggest a beneficial effect of capsaicin intake on the lipid profile of the animals.

### 3.2. Capsaicin Reduces Inflammatory Markers in the Liver of ApoE KO Mice

**Figure 2:**
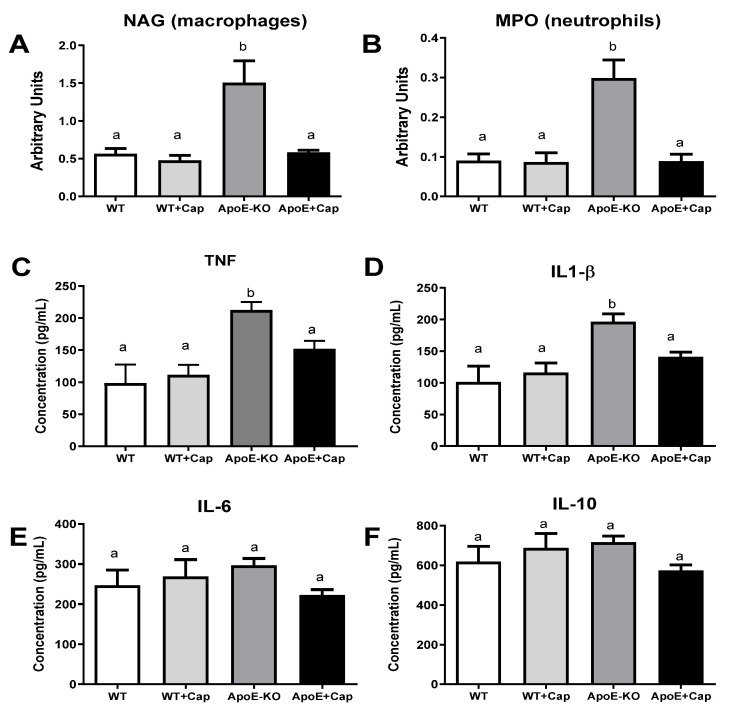
Modulation of Inflammatory Markers by Capsaicin in WT and ApoE KO Mice. Quantification of NAG (**A**), MPO (**B**), and the cytokines TNF (**C**), IL-1β (**D**), IL-6 (**E**), and IL-10 (**F**) in the liver tissue of wild-type animals (C57BL/6) and ApoE KO mice receiving control (AIN-93M + 0.075% cholesterol) or capsaicin-containing (AIN-93M + 0.075% cholesterol + 0.015% capsaicin) diets for 5 weeks. Data are presented as mean (bars) and standard error (vertical lines). Groups not sharing the same letter = statistically different (*p* < 0.05). *N* mice = WT = 5, WT + CAP = 6, ApoE − KO = 11, ApoE + Cap = 10, except for NAG and MPO *n* = 5–6 mice/group.

As expected, ApoE KO mice exhibited heightened inflammatory markers compared to WT mice, except for IL-6 and IL-10 (Figure 2A–F). Supplementing ApoE KO mice with Cap reduced macrophage and neutrophil activity (measured by NAG and MPO enzyme activity, respectively) and TNF and IL1β concentrations to levels comparable to those in WT groups (Figure 2A–F). These findings suggest that Cap effectively regulates the basal inflammatory state in ApoE KO mice, bringing it closer to that of non-inflamed WT mice.

### 3.3. Capsaicin Treatment Reduces Atherosclerosis and Inflammation in ApoE KO Mice

Since C57Bl/6 mice would not develop atherosclerotic lesions in the short period used in this study, atherosclerosis development was only evaluated in ApoE KO groups by quantifying fatty streaks in the aorta and more advanced lesions in the carotid artery.

**Figure 3:**
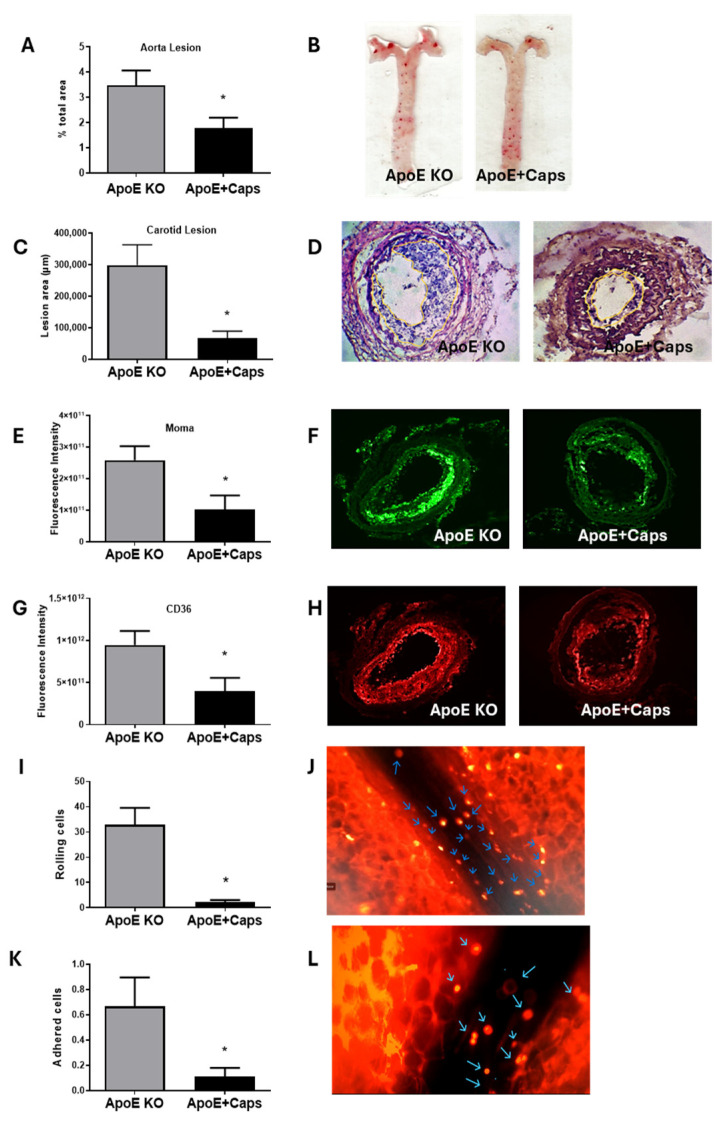
Effects of Capsaicin on Atherosclerosis Development and Inflammation (**A**,**B**) Percentage of the aorta affected by atherosclerotic lesions in ApoE KO mice after five weeks of ingesting a diet rich in cholesterol with or without Cap (0.015%) *n* = 8–9 mice/group (**C**,**D**)—Atherosclerotic lesion area in the obstructed carotid artery *n* = 7 mice/group. Fluoresce intensity of monocyte/macrophage (**E**,**F**) and CD36 (**G**,**H**) staining in the carotid artery (*n* = 7–10 mice/group). Rolling (**I**,**J**) and adhered (**K**,**L**) cells in the mesenteric plexus blood by intravital microscopy. Data are presented as mean (bars) and standard error (vertical lines). * Statistically different (*p* < 0.05). Blue arrows show rolling and adhering leukocytes. *N* mice = 6–8 mice/group.

Capsaicin treatment significantly reduced the area affected by a fatty streak in the aorta, especially in the aortic arch (Figure 3A,B). Similarly, the carotid presented smaller lesions in the ApoE + Cap group compared to the ApoE KO group (Figure 3C,D). These findings suggest that Cap has an atheroprotective effect, as chronic intake effectively delays the development of both early-stage (fatty streak in the aorta) and more advanced (carotid lesions) atherosclerosis.

To investigate the role of macrophage-derived foam cells, we examined the presence of macrophages and their main scavenger receptor, CD36, in the lesion site. The results showed a reduced area of MOMA staining in lesions of Cap mice, indicating decreased monocyte/macrophage recruitment (Figure 3E,F). This was further confirmed by the lower density of CD36 in the same group (Figure 3G,H).

To determine if the observed improvements in the inflammatory state were systemic, we conducted an intravital microscopy experiment to observe the activation of circulating leukocytes in real-time. Investigating leukocyte rolling and adhesion via intravital microscopy is crucial, as these processes initiate inflammatory responses which are pivotal in atherosclerotic plaque development. The results revealed a significant reduction in the leukocyte rolling and adhesion in animals fed with Cap, suggesting a systemic anti-inflammatory effect of Cap (Figure 3I–L), Supplementary Videos S1 and S2. In summary, capsaicin treatment reduces atherosclerosis development, which is linked with decreased macrophage recruitment and CD36 expression in lesions, suggesting its role in inhibiting foam cell formation.

### 3.4. Capsaicin Reduces Foam Cell Formation by Modulating Macrophage Cholesterol Influx and Efflux Pathways

**Figure 4:**
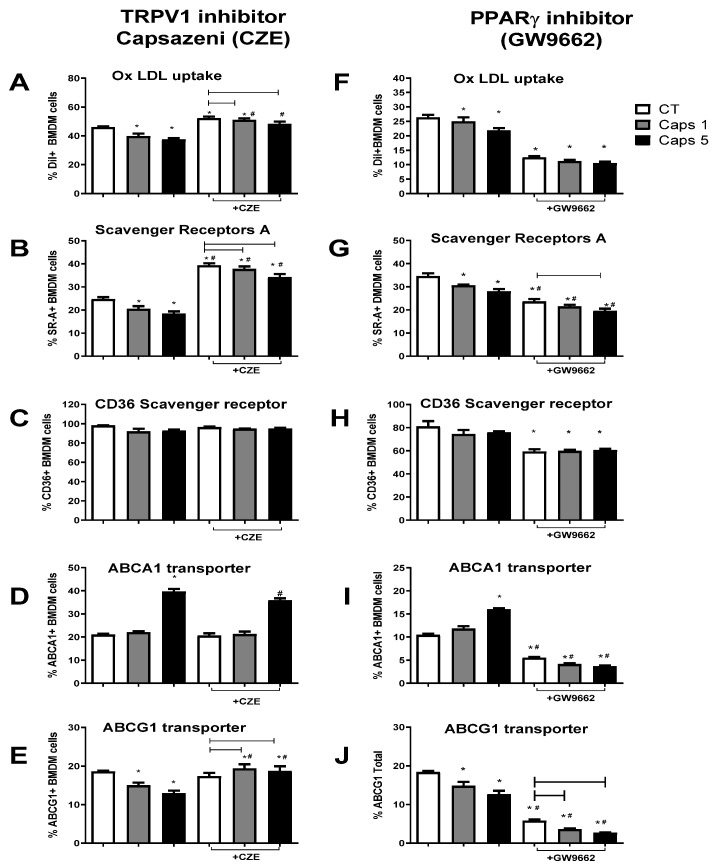
Cholesterol influx and efflux pathways in cultures treated without or with TRPV1 antagonist (CZE (**A**–**E**)) or PPARγ inhibitor (**F**–**J**). (**A**)—Total% of oxLDL-Dil positive populations treated or not with CZE; (**B**)—Total% of SRA-positive cells, treated or not with CZE; (**C**)—Total% of populations positive for CD36, treated or not with CZE; (**D**)—Total% of ABCA1-positive cells treated or not with CZE (**E**)—Total% of ABCG1-positive cells treated or not with CZE; (**F**)—Total% of oxLDL-Dil positive populations treated or not with GW9662; (**G**)—Total% of CD36-positive populations, treated or not with GW9662; (**H**)—Total% of SARS-positive cells, treated or not with GW9662; (**I**)—Total% of ABCA1-positive cells treated or not with GW9662; (**J**)—Total% of cells positive for ABCG1 treated or not with GW9662; Bars represent mean and vertical lines represent standard error (SE). Difference statistically significant (*p* < 0.05) is provided as (*) between each group (with or without inhibitors) and the control group (CT) without inhibitors; (—) between Caps groups treated with inhibitors and the CT group treated with inhibitors; (#) between groups treated with the inhibitor and the same group without the inhibitor.

After demonstrating the anti-atherosclerotic effects, we evaluated the influence of Cap on macrophage-derived foam cells by measuring the changes in oxLDL-loaded cells, expression of the influx-related receptors (CD36 and SRA), and the efflux-related transporter (ABCA1 and ABCG1). In BMDM cells pretreated with 1 or 5 µM Cap without any inhibitor, foam cells (oxLDL-Dil ^+^ cells) were reduced compared with non-treated cells (Figure 4A–F). This effect was accompanied by fewer cells expressing the SRA scavenger receptors (Figure 4B–G) without altering the number of CD36-expressing cells (Figure 4G,H). Regarding efflux transporters, Cap treatment increased the proportion of cells expressing ABCA1 in a 5 mM concentration (Figure 4D–I). Interestingly, the number of cells expressing ABCG1 decreased with both concentrations of Cap. (Figure 4E–J). These results suggest that Cap reduces foam cell formation by lowering cholesterol influx via SRA downregulation and increasing ABCA1-mediated efflux.

We investigated the influence of the natural Cap ligand, TRPV1, by treating cells with the TRPV1 inhibitor CZE. While TRPV1 inhibition increased foam cell formation, Cap’s ability to reduce oxLDL uptake remained evident (Figure 4A). Confirming our previous data, foam cell increase was associated with increased SRA-expressing cells without effects on CD36-expressing cells (Figure 4B,C).

TRPV1 inhibition, however, did not affect ABCA1-expressing cells in control or 1 µM Cap-treated cells (Figure 4D), although a slight reduction of ABCA1 was seen with 5 µM treatment. Regarding the ABCG1 receptor, CZE treatment increased this transporter only in capsaicin-exposed cells, suggesting TRPV1 inhibits Cap’s action on ABCG1 expression. Together, these data indicate that Cap’s action in reducing cholesterol influx (via SRA) and ABCG1 is dependent on TRPV1, although the action on the ABCA1 transporter is independent of TRPV1.

Given that TRPV1 does not influence ABCA1, we investigated another target of capsaicin signaling, the PPARγ receptors. Our results showed that the inhibition of PPARγ, unlike what was seen with the TRPV1 inhibition, drastically reduced the foam cells in control and Cap-treated cells (Figure 4F) and was associated with the decreased cells expressing SRA and CD36 (Figure 4G,H). Nonetheless, Cap does not affect the CD36 receptors since there is no difference between Cap and the control groups. Regarding SRA, Cap has a small but significant effect at 5 µM after PPARγ inhibitor treatment.

When evaluating efflux transporters, PPARγ inhibition reduced the expression of ABCA1, abolishing the effect of the Cap (Figure 4I). However, the expression of ABCG1, although significantly reduced in CT cells, maintains the pattern seen in Cap cells without GW9662 treatment, showing a reduction in the number of cells expressing this transporter (Figure 4J). These results suggest that PPARγ promotes influx receptor expression and foam cell formation, while its inhibition drastically inhibits CD36 and SRA. Regarding efflux transporters, the increase of ABCA1 expression related to Cap treatment is dependent on PPARγ. Conversely, the ABCG1 expression seems to be favored by PPARγ activation, although the effect of Cap in reducing this transporter is independent of it.

## 4. Discussion

Our study demonstrated that capsaicin effectively reduces systemic inflammation, dyslipidemia, and atherosclerosis development in ApoE KO mice. The observed decrease in visceral adipose tissue, independent of food intake, aligns with existing literature on capsaicin’s metabolic effects, enhanced lipolysis, and stimulation of browning [9,10,23,24]. Visceral fat is an important trigger of inflammation, and its reduction is crucial to improving the inflammatory state observed in both obesity and atherogenesis [9].

Hypercholesterolemia, particularly elevated levels of non-HDL cholesterol (atherogenic fraction) and, to a lesser extent, hypertriglyceridemia, are well-established risk factors for atherosclerosis. Our study, consistent with previous research, demonstrated an improvement in the lipid profile following capsaicin supplementation in several animal models and clinical trials, suggesting that capsaicin is a promising approach for reducing circulating levels of LDL-C in patients with metabolic syndrome [25].

Atherosclerosis involves the uptake of modified oxidized apo B-containing lipoproteins (non-HDL) by macrophages and smooth muscle cells (SMCs). This leads to foam cell formation via influx scavenger receptors such as SRA and CD36. Cholesterol efflux, regulated by ATP-binding cassette (ABC) transporters such as ABCA1 and ABCG1, is crucial for maintaining intracellular cholesterol homeostasis. ABCA1 facilitates the transfer of cholesterol and phospholipids to apoprotein A–I, forming nascent HDL, while ABCG1 promotes cholesterol efflux from macrophages to HDL [26]. Our study found that reducing non-HDLc while maintaining HDLc levels in the ApoE + Cap group was critical for delaying atherosclerosis progression in the aorta and reducing advanced lesions in the carotid artery.

The reduction of visceral adiposity, blood lipids, and atherosclerosis lesions, prompted us to investigate whether systemic inflammation was also diminished. Some inflammatory markers, including NAG, MPO, TNF, and IL-1β were significantly decreased. Notably, the reduction in MPO activity indicates not only an improvement in inflammation but also in oxidative status, as MPO activity involves reactive oxygen species (ROS) generation. MPO also contributes to chemical modifications, which increases foam cell formation [27].

High concentrations of TNF and IL-1β compromise the normal functioning of endothelial cells (EC), inducing oxidative stress, producing procoagulant factors, and impairing vasodilation, accelerating atherothrombosis [28]. Cap’s reduction of hepatic inflammation could depend on TRPV1 activation because the reduced serum levels and hepatic expression of TNF and IL-1β in LPS-treated mice were not observed in TRPV1 knockout mice [29]. This effect was associated with the release of calcitonin gene-related peptide (CGRP), resulting from the activation of TRPV1.

To explore the systemic repercussions of capsaicin’s anti-inflammatory effects, we used intravital microscopy to quantify leukocyte rolling and adhesion. The adherence of leukocytes to the vascular endothelium is a striking feature of the inflammatory process. Initial adhesive interactions between leukocytes and the endothelium involve tethering (capturing) and rolling. This initial weak interaction may become more robust by leukocyte activation, leading to stronger adhesion. These cells can then migrate into the interstitium through spaces between adjacent endothelial cells (ECs), initiated by various chemical mediators from the inflamed tissue [30]. Our study demonstrated that leukocyte rolling, and adhesion were reduced in animals fed with capsaicin, reinforcing the anti-inflammatory effects of this compound shown in the liver, aorta, and carotid. Schneider et al. [31] obtained similar results using intravital microscopy to explore the role of CGRP in acute pancreatitis in Wistar rats. The authors demonstrated that a capsaicin injection before pancreatitis induction could reduce tissue damage and 24-h mortality from pancreatitis by improving perfusion and reducing pancreatic inflammation.

Although our in vivo study highlighted the atheroprotective action of capsaicin, it also has some limitations. Firstly, the surgical carotid obstruction may not accurately replicate the natural development of atherosclerosis in humans, as it may not fully capture the complexity and variability of the disease process. Additionally, Apo E KO mice do not exhibit a lipoprotein profile identical to that of humans. This disparity can lead to significant differences in the progression and response to treatment of lipid-related diseases, such as atherosclerosis, which are closely linked to lipoprotein metabolism. Nonetheless, these limitations underscore the importance of complementing animal studies with additional research, using models or systems that more closely resemble human physiology or directly in human populations to ensure the relevance and applicability of the findings.

Capsaicin has been shown to reduce the inflammatory response by regulating intracellular lipid overload [20,32,33]. However, there is no consensus on the exact mechanisms mediating this process. Some studies suggest that Cap minimizes cholesterol load and inflammation in foam cells independently of TRPV1 [20,32,33], potentially by regulating cholesterol efflux via LXR-α and PPARγ [25,34]. In contrast, other studies demonstrate that TRPV1 signaling is essential for Cap to prevent foam cell formation [29].

Our experiments revealed that Cap pre-treatment reduces LDL-loaded foam cells by modulating the expression of influx and efflux receptors through pathways involving TRPV1 and PPARγ. Cap significantly reduced the expression of SRA, dependent on the TRPV1 activation. Inhibition or deficiency of TRPV1 abolished this reduction, while PPARγ inhibition decreased SRA-expressing cells. This PPAR-mediated action was previously observed in SMC-derived foam cells, where PPARγ expression induces CD36 expression and subsequent lipid accumulation [35,36]. Our results in BMDMs align with these findings, showing reduced CD36 and SRA expression following PPARγ inhibition. Although we did not measure the expression of PPARγ target genes to confirm the inhibitory effect of GW9662 in our experimental model, our study demonstrated that this antagonist influenced the expression of both efflux and influx receptors in BMDM cells, with significant alterations observed in both control and Caps-treated cells. Supporting our results, Martin-Fuentes et al. [37] showed that CD36 expression in macrophages derived from human monocytes incubated with ox-LDL is mainly regulated by pro-inflammatory cytokines, while SR-A expression correlates with PPARγ and NF-kB. Thus, PPARγ activation by capsaicin in the absence of functional TRPV1 increases in SRA-expressing foam cells.

In the presence of TRPV1, as observed in BMDMs without antagonism, Cap reduced the foam cell population modestly (approximately 13%) but significantly, suggesting that TRPV1 activation overrides the PPARγ effect of increasing SRA. Additionally, treating cells with PPARγ inhibitors highlights the role of TRPV1 activation in reducing SRA and CD36 expression, as neither Cap nor oxLDL alone effectively induced the expression of influx receptors, leading to a significant reduction in foam cell population.

Our study is innovative as it covers not only the risk factors for atherosclerosis but also the characterization of biomarkers for the development of both early and advanced lesions. It highlights the importance of antioxidants and, especially, the anti-inflammatory action of capsaicin in this process. The study also proposes a mechanism related to the formation and modulation of foam cells in macrophages. Therefore, our results make significant contributions and pave the way for clinical studies.

## 5. Conclusions

In conclusion, capsaicin demonstrates significant potential in reducing atherosclerosis progression by modulating cholesterol metabolism and inflammation while inhibiting foam cell formation. Also, our data suggest that Cap effectively reduces oxLDL-loaded foam cells by downregulating the scavenger receptor SRA and increasing the efflux transporter ABCA1. Importantly, these effects are partially mediated by TRPV1 and independent of PPARγ activation. In the absence of functional TRPV1, Cap exerts a more modest effect on foam cell reduction. PPARγ activation by Cap in vitro reduces cholesterol efflux and increases influx scavenger receptors. Mechanistic insights into capsaicin’s actions involving TRPV1 and PPARγ pathways provide further rationale for exploring its clinical applications in managing atherosclerosis and related complications.

## Data Availability

The datasets analyzed during the current study are available from the corresponding author upon reasonable request.

## Supplementary Materials

The following supporting information can be downloaded at: https://www.mdpi.com/article/10.3390/nu16183167/s1, Supplementary Video S1, and Supplementary Video S2.
